# *INPP5D*/SHIP1: Expression, Regulation and Roles in Alzheimer’s Disease Pathophysiology

**DOI:** 10.3390/genes14101845

**Published:** 2023-09-23

**Authors:** Edward O. Olufunmilayo, R. M. Damian Holsinger

**Affiliations:** 1 Laboratory of Molecular Neuroscience and Dementia, School of Medical Sciences, Faculty of Medicine and Health, The University of Sydney, Camperdown, NSW 2050, Australia; eddietobie@gmail.com; 2 Department of Medicine, University College Hospital, Queen Elizabeth Road, Oritamefa, Ibadan 2002012, Nigeria; 3 Neuroscience, School of Medical Sciences, Faculty of Medicine and Health, The University of Sydney, Sydney, NSW 2006, Australia

**Keywords:** SHIP1, Alzheimer’s disease risk gene, single-nucleotide polymorphism, microglia

## Abstract

Alzheimer’s disease (AD) is the most common form of dementia, accounting for approximately 38.5 million cases of all-cause dementia. Over 60% of these individuals live in low- and middle-income countries and are the worst affected, especially by its deleterious effects on the productivity of both patients and caregivers. Numerous risk factors for the disease have been identified and our understanding of gene–environment interactions have shed light on several gene variants that contribute to the most common, sporadic form of AD. Microglial cells, the innate immune cells of the central nervous system (CNS), have long been established as guardians of the brain by providing neuroprotection and maintaining cellular homeostasis. A protein with a myriad of effects on various important signaling pathways that is expressed in microglia is the Src Homology 2 (SH2) domain-containing Inositol 5′ Phosphatase 1 (SHIP1) protein. Encoded by the *INPP5D* (Inositol Polyphosphate-5-Phosphatase D) gene, SHIP1 has diminutive effects on most microglia signaling processes. Polymorphisms of the *INPP5D* gene have been found to be associated with a significantly increased risk of AD. Several studies have elucidated mechanistic processes by which SHIP1 exerts its perturbations on signaling processes in peripheral immune cells. However, current knowledge of the controllers of *INPP5D*/SHIP1 expression and the idiosyncrasies of its influences on signaling processes in microglia and their relevance to AD pathophysiology is limited. In this review, we summarize these discoveries and discuss the potential of leveraging *INPP5D*/SHIP1 as a therapeutic target for Alzheimer’s disease.

## 1. Introduction

Alzheimer’s disease (AD) is a common progressive neurodegenerative disease typically affecting individuals over the age of 65. It accounts for approximately 70% of all-cause dementia. Recent statistics have revealed that approximately 55 million individuals are living with dementia worldwide [1]. Cognitive decline and neuropsychiatric symptoms cause deterioration in quality of life and the need for 24-hour care, culminating in serious personal impact on patients and caregivers. Dementia, therefore, is an enormous global health burden, and the development of effective therapies is critical to effectively managing AD.

Genetic predisposition is an important AD risk determinant, with heritability estimates of 60–80% [2]. Microglia are the predominant immune cells in the brain, and genes exclusively or highly expressed in microglia, such as TREM2, CD33, PLCG2, ABI3 [3] and INPP5D [4], have been found to exert various influences on the development of AD.

Central nervous system (CNS)-resident microglia provide innate immune defense against infection and trauma and maintain tissue homeostasis by performing synaptic pruning and clearing apoptotic cells and cellular debris. The blood–brain barrier prevents the adaptive immune system from playing a significant role in the CNS by excluding peripheral adaptive immune cells from the brain, although new evidence suggests that antigens and antigen-presenting cells originating from the CNS have been found in the peripheral lymphatic system and may stimulate adaptive and humoral immune reactions [5]. Once activated from their homeostatic or quiescent state, microglia assume a ramified morphology and efficiently clear various cellular and acellular material that has been marked for disposal. Such materials are generally produced by a variety of insults such as acute damage caused by trauma, or ongoing damage, such as that characteristic of chronic neurodegenerative diseases, including AD. In AD, these damage-related materials and signals arise from plaques, fibrils and insoluble aggregates of amyloid-β (Aβ), damaged myelin, extracellular matrix, and stressed as well as degenerating neurons [6].

As AD develops, microglia lose their innate function and form clusters surrounding amyloid plaques and other damage signals [7]. Either through local proliferation or migration, microglia alter their morphology to become more amoeboid as they transform into a reactive state and prime their protein-synthesizing machinery to express various proteins needed to mount an immune response and, importantly, promote phagocytosis [7].

In 1994, Saxton and colleagues [8] reported that a 145 kDa protein became phosphorylated and associated with the adaptor protein Shc1 following B-cell or cytokine receptor stimulation. Shortly thereafter, the 145 kDa protein was identified by a number of research groups as Src Homology 2 (SH2) domain-containing Inositol 5′ Phosphatase (SHIP) [9,10,11], a protein encoded by the gene *INPP5D* (a member of the inositol polyphosphate-5-phosphatase) at the locus 2q37.1. Polymorphisms of *INPP5D* have been associated with higher risk of late-onset AD (LOAD) [4]. The single-nucleotide polymorphisms (SNPs) rs35349669 and rs10933431 in the *INPP5D* gene were significantly associated with increased AD risk [12]. *INPP5D* is associated with another major AD risk gene—*TREM2* (Triggering Receptor Expressed on Myeloid cells 2). It inhibits TREM2 signaling through its interactions with DAP12 (DNAX-activating protein of 12 kDa), an adaptor protein that is important for TREM2 signaling and function [13]. Thus, SHIP1 potentially plays important roles in the pathophysiology associated with Alzheimer’s disease, and therefore, numerous small-molecule inhibitors of SHIP1 protein are currently being studied as possible immune-based therapies for AD. In this review, we aim to discuss the current knowledge of processes driving *INPP5D* transcription, the actions of its gene product SHIP1 in microglia and their relevance to AD, while examining its potential as a novel therapeutic target for Alzheimer’s disease.

## 2. Expression and Regulation of *INPP5D* in AD

*INPP5D* is a gene that is advancing to the forefront of genetics research in AD as a result of one of its SNPs, rs35349669, being associated as a common variant of the disease, accounting for 3.8% of all genetic risk for AD [14].

*INPP5D*, expressed in the brain, mostly in microglia, typically includes a 27-exon gene that encodes several isoforms. The full-length 27-exon isoform encodes an amino-terminal SH2 domain followed by a phosphatase domain, while the transcription of truncated isoforms lacking the SH2 domain originates from internal transcription start sites (TSS) [12].

The minor intron 10 allele of rs35349669 is associated with increased risk of AD, while the minor allele of rs10933431 located in intron 2 is associated with decreased AD risk [4,15]. According to Zajac et al. [12], isoforms of *INPP5D* associated with TSSs located in exon 1 and intron 14 are markedly increased in individuals with high AD neuropathology. In addition, expression of a novel variant (referred to as the D47 variant) that lacks a 47 bp segment from exon 12 was also increased in AD brains, resulting in ~13% of total *INPP5D* expression, and was found to undergo nonsense-mediated decay [12]. The missing 47 base pairs results in a frameshift and a consequent introduction of a premature termination codon (PTC), with the resulting protein being devoid of a phosphatase domain. The rs35349669 SNP correlates with an allele-specific expression of full-length *INPP5D* [12].

The identified TSS of *INPP5D* includes TSS-A in the SH2 domain, and TSS-B and TSS-C in the phosphatase domain, all of which result in the production of different SHIP1 isoforms. TSS-A is located in exon 1 and generates isoforms 201, 204 and 205, whilst TSS-B originates in exon 11, producing isoform 202, and TSS-C is located in intron 14 and generates isoform 213. They also showed that isoforms originating from TSS-A and TSS-C are upregulated in the AD brain compared to the isoform produced by TSS-B. They concluded that individuals with AD may have an altered transcription factor profile or open chromatin sites that influence access to the TSS of the *INPP5D* gene, which in turn promotes transcription of various SHIP1 isoforms, leading to the propagation of AD pathology [12]. The Ikaros family of genes encodes for five zinc-finger proteins—Ikaros (*IKZF1*), Helios (IKZF2), Aiolos (*IKZF3*), Eos (*IKZF4*) and Pegasus (*IKZF5*)—that act as transcription factors, exerting critical regulatory roles during the development and function of various immune cells [16,17]. Ikaros exerts a crucial regulatory role in B-cell receptor (BCR) signaling, as studies have found that disruption of Ikaros in the chicken B cell line DT40 results in a diminution of BCR signaling, with reduced PLCγ2 phosphorylation and impaired intracellular calcium mobilization and signaling [18,19]. Other studies have revealed that Helios exerts a directly opposite effect as its knockout resulted in elevated BCR signaling [20]. Both Ikaros and Helios bind similar DNA sequences and chromatin remodeling complexes in the upstream regulatory region of the *INPP5D* gene to exert their effects as transcription factors [21,22].

Studies by Tsai and colleagues [23] revealed significantly elevated *INPP5D* expression in human AD microglial cells, and demonstrated a direct correlation between increased *INPP5D* expression and amyloid deposition in specific brain regions, including the inferior frontal, parahippocampal and superior temporal gyri, as well as in the frontal pole, although they did not delve into mechanisms driving specific *INPP5D* gene expression. There have been attempts to explain the increased expression of *INPP5D* in AD, where it has been proposed to be related to neuroinflammation and amyloid-plaque-associated increase in the expression of the transcription factor PU.1, which has been strongly associated in AD genetics. PU1 has been shown to bind to and upregulate *INPP5D* expression as it does other AD-related genes including TYROBP, MS4As, TREM2, and CD33 [24]. However, more studies are required to characterize the mechanisms that underpin the increase in *INPP5D* expression in AD, how this might be mitigated, and if such manipulations may be of any tenable therapeutic benefits.

## 3. SHIP1: Structure

Saxton and colleagues [8] were studying antigen-induced B-cell signaling and Ras activation when they discovered that AgR-induced signaling stimulated phosphorylation of the adapter protein SHC and mSOS1. This in turn resulted in the formation of membrane-associated complexes composed of SHC, GRB-2, mSOS1, and a previously uncharacterized 145 kDa protein [8]. Chacko et al. [9] subsequently identified the protein to be Src homology 2 (SH2) domain-containing inositol 5′ phosphatase 1 (SHIP1). Their work revealed that phosphorylation of SHIP1 and its association with Shc required co-clustering with the Fc receptor for IgG (FcγRIIB) and not stimulation of the B-cell receptor (BCR) alone. Since it was previously discovered that co-clustering of the BCR with FcγRII reduced proliferation induced by the stimulation of the receptor, they concluded that tyrosine phosphorylation of SHIP1 and its association with Shc resulted in negative signaling through effects on phosphatidylinositol metabolism [9]. SHIP1 exerts its effects on FcγRIIB by acting at its immunoreceptor tyrosine-based inhibitory motif (ITIM) [25].

**Figure 1:**
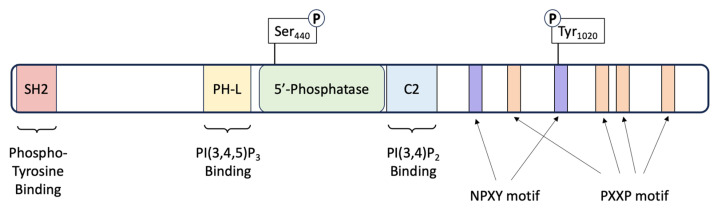
Schematic of the SHIP1 protein. SHIP1 is a 145 kDa protein consisting of 1189 amino acids. The protein consists of several domains that impart various functionalities. A central catalytic domain (5’-Phosphatase) is flanked by a Pleckstrin homology-like (PH-L) and a C2 domain for binding of the substrate PI(3,4,5)P3 and product PI(3,4)P2 of the enzyme. The carboxy-terminus of the protein contains four Proline-rich (PXXP) motifs, important for SRC Homology 3 (SH3) binding. The C-terminus also harbors two Asn-Pro-X-Tyr (NPXY) motifs. The protein also contains two phosphorylation sites: one within the phosphatase catalytic domain at Serine 440, and the second on a tyrosine residue at amino acid 1020 located within the second NPXY motif.

SHIP1 is a 1189-amino-acid protein with several splice variants that have multiple distinct motifs and domains that contribute to its numerous cellular functions [26]. The functionally important domains include the phosphatase domain, SRC-Homology 2 (SHC) domain, Pleckstrin Homology-related (PH-R) domain and the C-terminus [25] (Figure 1).

The phosphatase domain of SHIP1 shares a 64% similarity to that of SHIP2, spans amino acids 401–866 and is responsible for its catalytic activity as a lipid phosphatase [27]. There are two allosteric activation sites located on either side of the phosphatase domain; a phosphorylation site at serine 440 and a C2 domain spanning amino acids 725–863 [25]. These sites are structurally and functionally important, as works by Zhang et al. [28] showed that the phosphatase activity of SHIP1 can be enhanced by protein kinase A-mediated phosphorylation at Ser440. In addition, the activity of this same domain may be increased by the direct binding of PI(3,4)P2 (phosphatidylinositol 3,4-bisphosphate) to the C2 domain [29].

The SH2 domain of SHIP1 shares 54% homology with that of SHIP2 and spans amino acids 5–101. Interaction of the SH2 domain with the intracellular components of various cell surface receptors is a critical mechanism that governs the localization of SHIP1, and therefore controls access to its substrate, PIP3 [27]. Known targets to which the SH2 domain binds include the ITIM (Immunoreceptor Tyrosine-based Inhibitory Motif) receptors FcγRIIB, gp49B1 and CD31, as well as the ITAM (Immunoreceptor Tyrosine-based Activation Motif) receptors FcγRIIA, FcγRI-associated zeta-chain, TCR zeta chain, FcεRI and the BCR [30]. Phosphorylated cytoplasmic proteins including Shc1 and Dok-3 can also interact with the SH2 domain of SHIP1 and compete with the aforementioned receptors for SHIP1 binding, or act as co-ligands in order to relay signals downstream [31,32]. The SH2 domain also interacts with PIP3 (phosphatidylinositol (3,4,5)-trisphosphate) and PI(4,5)P_2_ (phosphatidylinositol 4,5-bisphosphate) on the cell membrane, ensuring SHIP1 stability and presumably contributing to SH2-mediated recruitment of SHIP1 [33].

The PH-R domain of SHIP1 folds independently and spans amino acids 292–401, which also interacts with phosphoinositides, preferencing PIP_3_ [25]. Ming-Lum and colleagues [34] found that this domain was important for localization of SHIP1 to the phagocytic cup in macrophages.

The C-Terminus of SHIP1 varies from that of SHIP2 in that it contains several protein–protein interaction domains that are predicted to contribute to the regulation of adaptor and catalytic activities associated with this protein [25]. Two tyrosine residues within this region (Y915 and Y1022) mediate interaction with Shc1 [35], Dok-1 [36] and Dok-3 [30]. Mutation of these residues was shown to significantly reduce SHIP signaling in murine cells [35], thus underpinning their importance to the functions of the protein.

## 4. Actions of SHIP1 in Immune Cells, Including Microglia

**Figure 2:**
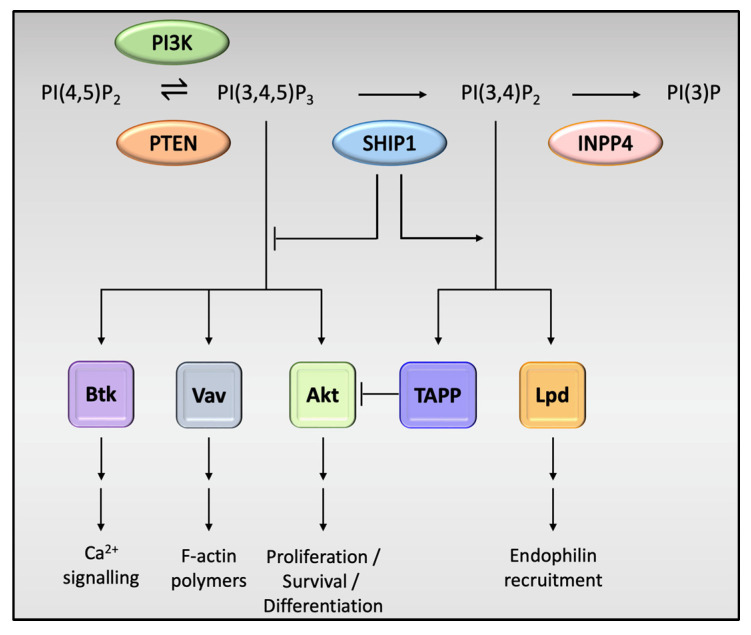
Phosphatase-dependent effects of SHIP1 on cell activity. PI(3,4,5)P3 is an activator of downstream signaling pathways, the most notable being the protein kinase Akt, which activates downstream signaling pathways required for cell growth, survival and proliferation. Other notable pathways include those involving Bruton’s tyrosine kinase (Btk), important in calcium signaling and the Vav family of proteins, which coordinate F-actin polymerization, supporting the cell cytoskeleton and structure. SHIP1 plays a pivotal role in the dephosphorylation of PI(3,4,5)P3, which in turn has an inhibitory effect on these pathways. The product of PI(3,4,5)P3 dephosphorylation, PI(3,4)P2, binds the tandem PH domain-containing protein (TAPP) adaptor proteins, which in turn inhibits Akt, further reducing the regulatory effects of this pivotal protein kinase. PI(3,4)P2 also enhances the recruitment of Lamellipodin (Lpd), an important regulator of cyto-skeletal assembly and cell migration.

SHIP1 is expressed in mesenchymal cells and plays a key role in the regulation of the immune system [37], while SHIP2 is ubiquitously expressed, and is mostly known for its effects on the regulation of blood glucose and metabolism [38]. Kerr [37] noted that these disparities may be due to differences in tissue distribution that translates to association with different receptors, unique actions on other signaling molecules and kinetic differences in binding to their PIP substrates. The effects of SHIP1 on signaling in immune cells are mediated by either phosphatase-dependent or phosphatase-independent mechanisms. SHIP1, via its enzymatic phosphatase domain, acts to dephosphorylate the inositol ring of PI(3,4,5)P_3_ at position 5 to generate PI(3,4)P_2_, thus preventing the recruitment of PI(3,4,5)P_3_-binding effector proteins such as Btk (Bruton’s tyrosine kinase) and Vav (Figure 2) [25]. Binding to PIP_3_ is crucial to the function of Btk [39]. Btk is a crucial regulator of PLCγ2 phosphorylation and controls Ca^2+^ fluxes in response to immune cell receptor activation [40]. SHIP1 inhibits intracellular Ca^2+^ responses by hindering the access of PIP_3_ to Btk, thus functionally impairing Btk. This negative effect on Ca^2+^ flux can also impair the activation of Ca^2+^-dependent downstream signaling effectors such as MAPK and NFκB, which have been shown by to be important downstream effectors in microglial TREM2 receptor activity [41]. Vav indirectly coordinates F-actin polymerization in immune cells by activating Rho-family GTPases and WASP, and its inhibition may potentially impair this function [42]. Loss of synaptic F-actin has been shown to be directly correlated with memory loss in AD [43]. This is a direct mechanism by which SHIP1 activity may contribute to progression in AD. However, SHIP1 also enhances the expression of adhesive integrin proteins in macrophages and microglia in a phosphatase-dependent manner, and this may prove to be an important step in the phagocytic function of these cells [44]. Contrastingly, the phosphatase activity of SHIP1 produces PI(3,4)P_2_, which enhances the recruitment of distinct PI(3,4)P_2_-binding effector proteins, such as TAPP1/2 and lamellipodin. Landego and co-workers [45] showed that binding of TAPP (tandem pleckstrin homology domain containing protein) adaptor proteins to PI(3,4)P_2_ exerts an inhibitory effect on the serine/threonine kinase, Akt. SHIP1 has previously been reported to inhibit the kinase activity of Akt, possibly via the mechanism described above [46]. Studies by Yao et al. [47] have shown that Akt plays an important role in downstream mechanisms initiated by TREM2 receptor activation in microglia, whereby SHIP1-mediated inhibition of Akt activity, albeit indirectly, may act to reduce TREM2-induced signaling in microglia. These activities may have potentially important implications for the pathogenetic processes involved in various neurodegenerative conditions.

SHIP1 also has effects on immune cells that are initiated by phosphatase-independent mechanisms, which serve to mediate or inhibit protein–protein interactions. One mechanism involves interaction of the C-terminal domain of SHIP1 with the PTB (protein tyrosine-binding) domain of Shc [48]. This interaction interferes with the binding of Shc to the Grb2-SOS complex, an association that generally results in the activation of Ras and the MAP Kinase pathway [49]. Binding of SHIP1 to Shc thus prevents activation of the MAPK pathway and ultimately signaling in immune cells. Another phosphatase-independent mechanism controlling immune cell signaling involves binding of the SHIP1 C-terminal domain to Dok-1. This causes localization of a Dok-associated RasGAP to the membrane, resulting in inhibition of Ras-Erk signaling [36]. The inhibition of this particular signal transduction system has been shown to reduce receptor-induced TNF–α generation in macrophages and microglia [50]. In monocytes and similar cells, SHIP1 inhibits NOD-2 complex-induced NFκB activation by blocking the binding of XIAP and RIP2 to NOD-2, preventing formation of this important complex [51], which can also impair signaling in microglia.

DAP12 is an ITAM, which associates with the transmembrane domain of the TREM2 receptor on microglia and is critical to the function of this receptor and activation of downstream signaling [52]. Acting via its SH2 domain, SHIP1 binds to DAP12 and inhibits signaling intermediates downstream of the TREM2-DAP12 complex, thereby reducing cellular activation, survival, actin reorganization, Ca^2+^ flux and cellular differentiation [13].

All the mechanistic effects of SHIP1 in immune cells, including microglia that have been described above, point to a uniform conclusion that SHIP1 activity tends to inhibit various receptor-induced signaling and resultant downstream mechanisms in these cells. The implications of this phenomenon for infectious, neoplastic and neurodegenerative conditions such as Alzheimer’s disease still remain to be studied and could potentially lead to the development of novel, effective therapies for these diseases.

## 5. Roles of SHIP1 in Alzheimer’s Disease Pathophysiology

The characteristic pathological changes observed in the AD-affected brain at autopsy include amyloid plaques, neurofibrillary tangles (NFTs), neuronal loss, neuronal dystrophy, amyloid angiopathy and neuroinflammation. Although the series of events leading to the final path of neurodegeneration remain unclear, the accumulation of amyloid in the form of plaques is widely accepted as a central pathological event in AD. Virtually all mutations associated with AD increase the generation of Aβ peptide. This protein is derived from the sequential cleavage of the membrane-associated amyloid precursor protein (APP) via the actions of BACE1 (β-site Amyloid Precursor Protein-cleaving Enzyme 1) and the gamma secretase complex of proteins. Aggregated species of Aβ are neurotoxic and form the core of neuritic plaques. Neuronal loss leads to neurochemical deficits that in turn lead to AD. The loss of cholinergic neurons in important regions such as the basal nucleus of Meynert, combined with the reduction in acetylcholine neurotransmitter levels, has been a well-established contributor to the cognitive deficits observed in AD. These pathological events disrupt cell-to-cell communication, result in the abnormal synthesis and accumulation of cytoskeletal proteins, contribute to the loss of synapses and exaggerated pruning of dendrites, cause neuronal damage through oxidative metabolism, and eventually lead to cell death [53]. SHIP1 has been shown to contribute via different pathways to many of these pathological events, potentially resulting in increased risk and severity of the disease.

The mutation that characterizes the Rs35349669 variant of *INPP5D* is located near an internal TSS that is critical for the transcription of the SH2 domain of SHIP1 [54]. This polymorphism results in the production of a SHIP1 transcript that lacks the SH2 domain. In *INPP5D*-haplodeficient mice models, Iguchi et al. [55] and Lin et al. [56] showed that downregulation of *INPP5D* resulted in an increased association of microglia with amyloid plaques. Additionally, they showed that *INPP5D* downregulation restored plaque compaction, and increased microglial motility towards and phagocytosis of amyloid plaques, with a resultant alleviation of cognitive deficits compared to mice expressing normal levels of *INPP5D*. This finding suggests that SHIP1 plays roles and exerts actions that generally tend to promote AD pathology. Similarly, studies by Castranio and colleagues [57] also demonstrated that downregulation of *INPP5D* increased the association of microglia with Aβ plaques, although these researchers reported that these events increased plaque burden in the animals. Further studies will be needed to address these discrepancies.

**Figure 3:**
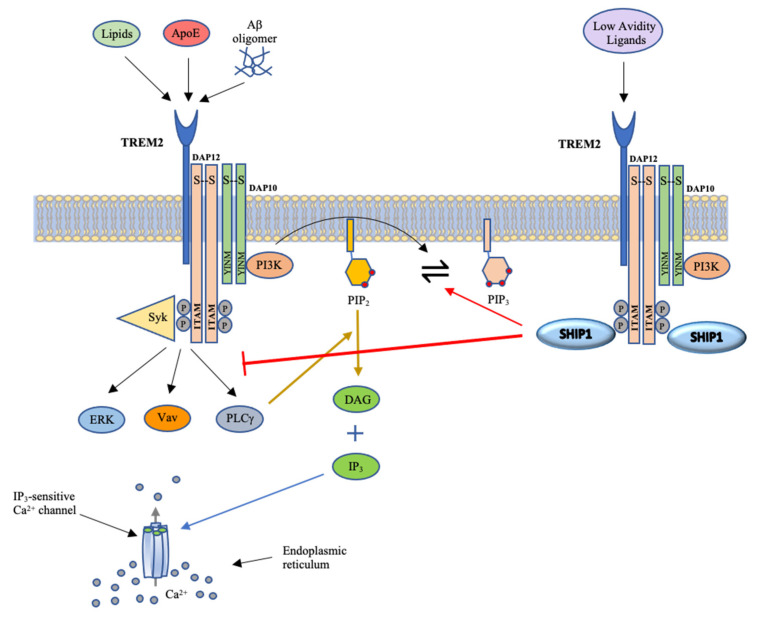
Schematic illustrating TREM2 signaling. The binding of ligands to TREM2 results in the phosphorylation of tyrosine residues within ITAM of DAP12 and the YINM motif of DAP10 by Src family kinases. This in turn forms docking sites for Syk and PI3K. PI3K converts PIP_2_ to PIP_3_, leading to cascades of signaling events. As outlined in Figure 2, these signals increase calcium flux and activation and nuclear localization of numerous transcription factors that promote cell proliferation, survival, phagocytosis, cytokine production and cytoskeletal rearrangement. The binding of SHIP1 to phosphorylated DAP12 facilitates the dephosphorylation of PIP_3_ and blocks the cascade of downstream signaling events. Image adapted from [6].

Knowledge of some of the actions of SHIP1 may be crucial to further understanding its influences in AD pathophysiology. SHIP1 has been shown to transduce inhibitory signaling of some ITIM-containing proteins and to inhibit signaling of ITAM-containing proteins such as DAP12 (Figure 3). SHIP1 thus tends to limit microglia-mediated inflammatory processes and phagocytic activities in AD [58]. SHIP1 has also been shown to display inhibitory effects on monocyte and microglia activation, partly by its action of transducing inhibitory signals from FcγRIIB and other ITIM-containing proteins [59]. Consequently, the response of microglia to detect and clear AD-associated damage-related substances such as amyloid plaques, insoluble Aβ aggregates, NFTs, extracellular matrix proteins and degenerating myelin and neurons may be grossly impaired. Also, as discussed earlier, the inhibitory effects of SHIP1 on the actions of the Vav protein in macrophages and microglial cells disrupt the processes involved in F-actin polymerization. This may result in impaired motility, microenvironment surveillance and phagocytic activity of these cells and can potentially contribute to or accelerate the molecular pathogenesis associated with AD. The depletion of PIP3 levels in these cells brought about by the actions of SHIP1 can also result in a blockage of the processes involved in microglial phagocytosis in response to the earlier mentioned AD-associated damage-related substances [60].

An interesting phenomenon mediated by SHIP1 that may be related to phagocytic processes is fast endophilin-mediated endocytosis (FEME) [61]. This pathway is independent of the widely known endocytotic pathway that utilizes clathrin-coated pits. Boucrot and colleagues [61] showed that in FEME, the PIP2 generated by the actions of SHIP1 on PIP3 recruits lamellipodin, which in turn recruits the protein endophilin. This process mediates ligand-triggered uptake of different receptors, cell-surface enzymes and even pathogens into the involved immune cell. The implications of this endocytotic pathway in AD pathophysiology require further investigation.

An additional function associated with SHIP1 relates to its role in regulating NF-κB (nuclear factor kappa-light-chain-enhancer of activated B cells), a protein complex that controls DNA transcription, the production of cytokines and cell survival. SHIP1 is a negative regulator of NF-κB. Exposure of postnatal day 2 astrocytes from SHIP1 knockout mice to TNF-α for 48 hours resulted in increased expression of NF-κB and a loss of the cytoplasmic inhibitor IκB alpha [62]. Similar results were observed in B and T cells from these knockout mice [63]. The ability of SHIP1 to regulate levels of NF-κB points to a critical role in the AD brain. Activation of NF-κB is known to increase expression of the AD-related β-secretase/BACE1 [64]. Whilst to our knowledge, levels of SHIP1 have not been measured in human AD brain, NF-κB levels, as measured by levels of its p65 subunit, are known to be significantly increased, particularly in the cortex, hippocampus and entorhinal cortex [65,66,67], frontal cortex [68] and basal forebrain [69]. Extending from these results, it could be inferred that SHIP1 levels are most probably decreased in the AD brain, indicating that, although numerous, microglial expression of SHIP1 is downregulated in AD. The reduction in SHIP1 would drive NF-κB activation, which in turn would lead to increased BACE1 in the AD brain. This results in the generation of more Aβ, which triggers further inflammation, thus setting up a vicious, positive feedback cycle as more NF-κB is produced due to inflammation.

Overall, the actions of SHIP1 in microglial cells in AD results in reduced effectiveness of the important functional processes of microglia (summarized in Table 1). These include, but are not limited to, microenvironment surveillance, detection of damage-related substances, ability to mount appropriate responses to these substances, maintenance of cytoskeletal physiology and framework and appropriate phagocytic processes, potentially leading to the accumulation of these toxic products and worsening of disease phenotype. These suggest that targeting SHIP1 with a therapeutic agent may be a viable option of novel treatment for Alzheimer’s disease.

## 6. SHIP1 as a Therapeutic Target for Alzheimer’s Disease

SHIP1 inhibition in microglia, potentially via the upregulation of TREM2 signaling as well as other mechanisms that have been discussed, may lead to a reduced risk of AD. SHIP2 expression has also been shown to be highly correlated with amyloid burden and cognitive decline in AD patients [70].

Obst and colleagues [71] performed crystallographic fragment screens of 597 compounds, and noted that the SHIP1-specific inhibitor 3AC increases downstream signaling following TREM2 stimulation, as evidenced by increases in SYK phosphorylation and intracellular Ca^2+^ flux. This suggests that SHIP1 inhibitors may assist microglia in harnessing the beneficial effects triggered by TREM2 activation in microglia and may be useful as therapeutic options for AD.

The first attempt at investigating whether inhibiting the activity of a SHIP protein could be used as a therapeutic modality was performed using AS1949490, a SHIP2 inhibitor. Administration of the compound to db/db mice, a model for type 2 diabetes, significantly lowered plasma glucose and improved glucose intolerance that is characteristic of these mice [72]. Administration of AS1949490 SHIP2 transgenic mice reversed memory deficits and improved synaptic plasticity and memory formation in db/db mice [73].

Pedicone and colleagues [74] investigated the effects of a number of selective SHIP1 inhibitors, selective SHIP2 inhibitors and pan-SHIP1/2 inhibitors on microglia and in mouse models of AD. Importantly, the agents tested were shown to easily penetrate the blood–brain barrier when administered orally or via the peritoneum, an important consideration when considering drug delivery to the CNS. In particular, a pan-SHIP1/2 inhibitor, K161, was found to significantly increase microglial proliferation, facilitate microglial phagocytosis and apoptotic neurons and Aβ_42_ [74]. These are major functions of microglia that are crucial for maintaining homeostasis in the CNS but are perturbed in AD. These effects were also examined using SHIP1-specific and SHIP2-specific agents, but their effects were noted to be much less significant compared to those observed using the pan-SHIP1/2 inhibitors [74]. These results suggest that a dual inhibition of the two SHIP paralogs is necessary for maximal therapeutic benefit. This may be explained by the possibility of higher PI(3,4,5)P_3_ quantities produced by dual SHIP1/2 blockade at the plasma membrane of microglia, which in turn enhances the efficiency of phagocytosis when microglia encounter a target to be phagocytosed, as mediated by TREM2 or Dectin1 [74]. However, since studies such as those by Sala Figerio et al. [75] have emphasized that SHIP1 diminution can also be very important in AD progression, it may be reasonably inferred that SHIP1 agonism should also be considered as a possible therapeutic option for AD. It has been suggested that the stage of disease could be a determinant of whether SHIP1 antagonism or agonism should be deployed as a therapeutic strategy, with SHIP1 antagonism favored during the early stages of disease in order to enhance the homeostatic functions of microglia and agonists potentially employed late in the disease possibly to dampen the effects of dysfunctional microglia in disease progression. More recent works by Pedicone and colleagues [76] showed that K306, a SHIP1-selective agonist, suppresses induction of inflammatory cytokines and iNOS expression in microglia, reduces TNF-α production, and enhances the degradation of synaptosomes and apoptotic neurons by microglia, revealing novel SHIP1-regulated functions that may be of potential therapeutic benefits in various dementias. These studies demonstrate that the homeostatic function of microglia could be pharmacologically modulated employing small-molecule SHIP inhibitors and more recently, agonists, which could lead to potential therapies for AD.

The simultaneous inhibition of SHIP1 and -2 using pan-SHIP1/2 inhibitors may offer a unique opportunity to enhance the basal homeostatic function of microglia that could be leveraged for therapeutic purposes in AD and potentially other neurodegenerative conditions that are also characterized by similar perturbations in microglia function. Hence, pan-SHIP1/2 inhibitors represent a novel form of immunotherapy for Alzheimer’s disease. However, further research is required to investigate some of these pharmacological agents, including newer lines of inquiry into SHIP1 agonists and potentially chaperone them through the drug development pipeline.

## 7. Conclusions and Prospects

SHIP1 is a large functional protein that is encoded by the *INPP5D* gene and has been shown to have wide-ranging mechanistic effects on cellular signaling in immune cells, particularly microglia, the immune cells of the CNS. Microglial cells are known to play critical roles in maintaining cellular homeostasis within the CNS and are intimately involved during various phases of Alzheimer’s disease. *INPP5D* expression and SHIP1 activity have been shown to be important risk factors in the development of late-onset AD, as the various mechanistic actions of SHIP1 in microglia result in dampening of signaling, and consequently of some of the beneficial functions of these cells. Studies on the effects of transcriptional regulation of *INPP5D*, SHIP1 blockade and more recently SHIP1/2 agonists are currently underway, with most studies showing promise, as they point to the fact that modulating the expression of SHIP1 and its paralog, SHIP2, may significantly improve disease phenotype and eventually provide novel therapeutic options for Alzheimer’s disease.

## Figures and Tables

**Table 1:** Summary of the involvement of SHIP1 in AD pathophysiology.

*INPP5D*/SHIP1 Deficiency	Effect	Reference
*INPP5D* haploinsufficiency (Tyrobp-deficient TREM2 loss-of-function mouse)	Restored microglial association with plaques, partially restored plaque compaction and reduced phosphorylated tau(+) dystrophic neurites	[55]
*INPP5D* haploinsufficiency	Increased dense-core plaques, microglial association with plaques and uptake of Aβ	[56]
*INPP5D* downregulation	Increased microglial association with plaques, but increased plaque burden	[57]
SHIP1—transducing inhibitory signals from FcγRIIB and ITIM-containing proteins	Inhibitory effects on monocyte and microglia activation	[59]
SHIP1—NF-κB pathway	SHIP1 downregulation leads to increased NF-κB activation, and an increase in BACE1 expression in AD brain	[62,63,64,65,66,67,68,69]

## Data Availability

All data are presented in the article.
